# Formal comment: Diagnostic labelling of mental health in young people: Perspectives from Kenya, South Africa and Zimbabwe

**DOI:** 10.1371/journal.pmen.0000666

**Published:** 2026-07-24

**Authors:** Rachel Maina, Marinos Bomikazi Lupindo, Charmaine Natasha Nyakonda, Tara Carney, Charity Mamathuba, Christine Musyimi, Charity Mokgaetji Somo, Eleanor Chatburn

**Affiliations:** 1 Stavros Niarchos Foundation (SNF) Global Center for Child and Adolescent Mental Health, Child Mind Institute, New York, United States of America; 2 Brain and Mind Institute, Aga Khan University, Nairobi, Kenya; 3 Global Brain Health Institute, Trinity College Dublin, Dublin, Ireland; 4 Experimental Psychology Department, University of Oxford, Oxford, United Kingdom; 5 Mind Matters NPO, Sandton, South Africa; 6 Department of Innovation and Learning, Grassroot Soccer, Hanover, New Hampshire, United States of America; 7 Mental Health, Alcohol, Substance Use and Tobacco Research Unit, South African Medical Research Council, Cape Town, South Africa; 8 Department of Psychiatry and Mental Health, University of Cape Town, Rondebosch, South Africa; 9 Department of Psychology, University of Johannesburg, Johannesburg, South Africa; 10 Research Department, Africa Institute of Mental and Brain Health, Nairobi, Kenya; 11 Department of Psychology of Education, University of South Africa, Pretoria, South Africa; 12 Department of Psychology, University of Cambridge, England, United Kingdom; 13 Norwich Medical School, University of East Anglia, Norwich, United Kingdom; 14 Department of Psychology, Sport and Sensory Sciences, Anglia Ruskin University, Cambridge, United Kingdom; PLOS: Public Library of Science, UNITED KINGDOM OF GREAT BRITAIN AND NORTHERN IRELAND

## Introduction

Research is emerging that explores the impact of diagnostic labels on perceptions of mild to moderate mental health conditions [1,2] in low- to middle-income country (LMIC) settings [3]. A recent study of adult residents in the USA by Altmann and colleagues [4] highlighted both positive outcomes of diagnostic labelling, including increased empathy and support and negative outcomes, including perceived lack of agency. In this study, as will be used in this Formal Comment, diagnostic labelling is used in the context of concept creep and concept breadth [4]. However, this research [4] has focused on contexts that mostly focus on mental health in settings with higher resources than LMICs that are often characterized by largely clinical approaches, while nuanced cultural factors such as spirituality and communal relations often found in LMICs including in Africa, have not yet been fully explored [5]. As authors working in Kenya, South Africa, and Zimbabwe, we explore how cultural perceptions shape the understanding and labelling of mental health symptoms and their resulting impacts.

### What is the understanding and perception of diagnostic labels in many African contexts? How do people in these contexts understand symptoms of mental health?

Some more individualistic societies occasionally have mental health labels which sometimes focus on the self for example self-control and perceived symptom persistence as denoted by Altmann and colleagues [4]; suggesting a more Cartesian framework of the mind and self as being self-contained. In certain LMIC settings in Africa, the concept of personhood and well-being does not exist in such isolation [6]. People relate with each other based largely on social identity which is primarily defined by the interplay between social, spiritual, and individual dimensions [6], which, in our experiences, sometimes contributes to mental health labels and related perception. At the social level, identity in settings such as South Africa is often rooted in the philosophy of Ubuntu, which suggests that human essence is found through interconnection, embodied in the Zulu phrase umuntu ngumuntu ngabantu (which denotes that life is fundamentally rooted in mutual aid) (In supplementary section we discuss further the reconceptualization of mental health labels through the Ubuntu Lens) [6]. This and other identities are further shaped by spiritual factors, such as the role of ancestors, and individual factors, where the physical self is seen as a direct extension of one’s spirit. In some African contexts, this is illustrated by the belief that physical attributes like hair are deeply connected to a person’s well-being; consequently, if someone gains access to them, they could potentially exert spiritual influence or even cause harm [7]. This shapes how mental health conditions are commonly understood, how communities respond, and how help is sought [4,8], suggesting that the use of diagnostic labels that are fundamentally individualistic is not always contextually and culturally sensitive to some contexts given the lived collective realities of individuals within some African contexts.

### How does this cultural understanding shape labelling practices and responses to diagnostic labels?

In some LMIC settings, some moderate to mild mental health symptoms such as those of depression are associated with adverse situations such as economic hardships, community unrest and loss as opposed to biomedical explanations for their existence [9,10]. Interventions in these cases may consider material, spiritual or welfare support and psychological reframing of struggles towards acceptance [10]. Understandably so, this response to labelling is in contrast to Altmann and colleagues [4] findings where labels invite need for professional mental health treatment. Without a biomedical conceptualisation of the symptoms, people are less likely to seek care from formal health facilities; those who do tend to have prior interaction with the formal healthcare system [10].

In our observations, responses to diagnostic labels can potentially be shaped by demographic factors. Altmann and colleagues [4] did not capture this nuance in their study. Take, for example, young people in Kenyan urban populations who may use diagnostic labels such as ‘being depressed’ or ‘having anxiety’ as a close proxy to sadness and worry as a “shared language” to describe complex emotions related to everyday experiences rather than referring to a DSM-5 diagnostic label [9,11]. This shorthand vernacular may lead to a range of emotional and cognitive experiences, such as sadness, overthinking, and other experiences of overwhelm and/or upset, being clustered and communicated as feeling “depressed” and “anxious”, the purpose of which is to validate each other’s lived experiences. This reverse use can further lead young people to perceive words like anxiety and depression as “cool” and “trending” rather than reflecting their clinical meaning [9]. This implies that labelling in such cases, does not always prompt a need for professional help as similar studies to Altmann and colleagues [4] would suggest.

Young people may also rely on their own perceptions and cultural paradigms to interpret mental health experiences; not from lack of knowledge as per Altmann and colleagues [4] but from different cultural understandings of symptom meaning and appropriate responses [12]. Atilola [12] describes this “lack of agency” perspective, where individuals with an external locus of control view distress as externally imposed yet individually controllable, leading them to engage in traditional and spiritual approaches to addressing their symptoms. At the other end of the age range, when younger people pathologize normal stress responses as clinical conditions, community responses may become dismissive, limiting support for those whose symptoms do meet diagnostic criteria [1,13], in contrast to Altmann and colleagues [4] findings. In such cases they may experience or perceive stigma from adults and healthcare workers when they use such labels or try to access services [1,13]. They may be perceived as ‘attention seekers’ or ‘lacking discipline,’ and their symptoms dismissed as ‘pretence,’ ‘laziness,’ or ‘proving to be difficult’ [2]. Milder symptoms may not be recognised as mental health conditions at all [1]; sympathy is withdrawn as distress is attributed to ‘thinking too much’ [1]. Such negative responses, together with the normalisation of mild to moderate mental health conditions, lead young people to conceal their distress and cope alone; those who do seek help often do so in hiding [2]. In our experience in South Africa for example, young people can cope with such a response by formulating a language that they can use to create a safe space to communicate with their peers about their mental health needs and which their caregivers sometimes find difficult to understand.

In our experience (also see Real World Example 1 and 2 in **Supplementary Materials**), younger populations tend to prefer mental wellness activities that are framed around activities they already enjoy (play, sports, arts, fun etc) over formal technical screening ‘mental health’ programs. This observation is consistent with studies which suggest that young people seek well-being support while avoiding formal diagnosis and diagnostic labels of mental health conditions [14]. When labels are used in everyday talk, peers often respond with empathy and support; where peers lack familiarity with mental health or appropriate treatment, responses may be negative [13].

### How can labelling be reconceptualised in some African settings?

Mental health conceptualisation in certain African contexts is less clear-cut than some dominant diagnostic models; it draws on psychological, social, and medical understandings [9]. In some clinical practice contexts where clinical terminology is used without the social and cultural context in which distress is usually understood, it tends to lose meaning. This implies that we need flexible approaches to provide safe spaces to promote mental well-being and treatment as opposed to only focusing on traditional conventional mental health services. Flexible approaches as captured by the case examples under the supplementary materials section. In our observations, we have identified this gap and are bridging this by creating interventions that are accessible, grassroots driven, destigmatising mental health conditions, co-designed with young people and building a sense of community and connectedness. This work has indicated that in general, a greater understanding of symptoms without labelling conditions is being sought and appreciated by young people [15]. This suggest that organisations working with young people may find ‘psychologising’ experiences ineffective in favour of interventions targeting destabilising factors, the enabling environment, and strengths and approaches that enhance connection rather than highlighting difference, or “deficits” as diagnostic labels can sometimes do. In pursing such a stance, diagnostic labels are neither seen as a blessing or not as attributed by Altmann and colleagues [4], but rather as inconsequential in the goal of alleviating mental distress.

### What is the way forward for clinical practice, policy, and community focused initiatives?

Diagnostic labels have cultural underpinnings that necessitate reframing of clinical practice, policy, and research in Kenya, South Africa, and Zimbabwe. Young people, including those with lived experience, must be involved in decisions about assessment terminology, disclosure, and management, ensuring approaches align with their perceptions and context rather than imposing external frameworks. Research involving young people should incorporate their conceptualization and interpretation perspectives. Policy should consider local perceptions of diagnostic labels when addressing stigma and allocating resources, particularly in settings where mental health knowledge and services remain limited. Table 1 outlines further recommendations specific to these contexts.

**Table 1:** Recommendations regarding the use of diagnostic language for key stakeholders.

Recommendations for Researchers	
*Research design*	Need for more integrative approaches to investigating the understanding and treatment of mental health conditions, and promotion of mental health within diverse communities. Co-creation which is grassroots driven and grounded in enabling environments where research conceptualization, aims, evaluation, outcomes and intervention strategies emerge and are shaped by consultations and engagement with young people, young people with lived experiences (with mental health, trauma and other social determinants of mental health) and community stakeholders.
*Participants*	Ensure that participants are recruited across the lifespan as young people may experience labelling effects differently from adults.
*Research population*	Design studies exploring the mental health labels conceptualisations by young people. Young people have diverse needs, beliefs, and barriers to help seeking, and are often less likely to seek treatment; an attribute that would have probably been highlighted if the study included this population [4].
**Recommendations for Programme Leaders**	
*Content of mental health events or initiatives*	Design events around positively framed attributes and skills such as life skills, promotion of wellbeing, positive young people development, sports for development and asset-based community development, rather than a deficit focussed approach perpetrated by clinical labels.
*Disability inclusion*	Prioritize disability inclusion during programme design and implementation to ensure that programmes align with their lived experiences, which young people are likely to relate to rather than mental health labels.
*Holistic support*	Design mental health programmes that address other factors such as social determinants of health which affect young people’s well-being outcomes like cultural perceptions of labelling.
**Recommendations for other stakeholder groups**	
*Media*	Programmes on mental health conditions should be more integrative. An integrative model, for example biomedical and spiritual, increases accessibility to a larger audience and helps people understand how these conceptualisations can help us understand and better support those experiencing mental health related challenges, whether with diagnostic labels or not. It also creates an opportunity to explore a more integrative approach to help-seeking.
*Government and Policy Makers*	Rural communities are some of the most vulnerable communities due to high levels of poverty and adversity, however, they still are the most poorly resourced when it comes to mental health knowledge, resources and service delivery, partly because of increased levels of stigma. Their responses to Altmann and colleagues [4] vignettes would have varied based on lack of knowledge. Increased resource allocation to these areas is paramount to improving understanding of mental health conditions and support services available.
*Stigma*	Address multilevel, intersectional stigma as a critical component of mental health programming for young people to encourage help seeking behaviour while sensitive to labelling perceptions. From policy to programme level, more work is needed to address different kinds of stigma at different levels-especially important to include the community due to the high levels of interconnectedness in many of the settings in LMICs.
*Measurement*	Use mixed methods in research and evaluation. Qualitative data provides unique insights which cannot be captured using quantitative measures alone; even when thoughtful considerations are made to increase reliability of the quantitative measures [4]. The stories that young people share about their lived experiences and the impact of programs that reconceptualise mental health labels, are central to understanding why and how change occurs including the evolving perceptions of labels.

## Supporting information

S1 Text Real world example 1: Overcoming linguistic and cultural barriers to mental health through football: The work of Grassroot Soccer in Kenya, Ethiopia, Malawi, Nigeria, South Africa, Zambia, Zimbabwe, Scotland and United States of America(https://grassrootsoccer.org/mental-health/). Real World Example 2: The work of Mind Matters in South Africa (https://www.mindmattersnpo.org/). The Radical Reconceptualization through the Ubuntu Lens.
(DOCX)
