# Supplementary material for: Formal comment: Diagnostic labelling of mental health in young people: Perspectives from Kenya, South Africa and Zimbabwe
Source: PLOS Ment Health. 2026 Jul 24;3(7):e0000666. doi: 10.1371/journal.pmen.0000666 (PMC13399455; doi:10.1371/journal.pmen.0000666)
Supplement: S1 Text — Real World Example 2: The work of Mind Matters in South Africa (https://www.mindmattersnpo.org/). The Radical Reconceptualization through the Ubuntu Lens. (DOCX) [file pmen.0000666.s001.docx]

**Formal comment: Diagnostic labelling of mental health in young people: perspectives from Kenya, South Africa and Zimbabwe**

**Supplementary information**

Note: The following examples from organizations where co-authors work are presented as observational illustrations of how young people in certain African contexts navigate mental health support in the absence of formal diagnostic labelling.

1. **Real world example 1: Overcoming linguistic and cultural barriers to mental health through football: The work of Grassroot Soccer in Kenya, Ethiopia, Malawi, Nigeria, South Africa, Zambia, Zimbabwe, Scotland and United States of America(**[**https://grassrootsoccer.org/mental-health/**](https://protect.checkpoint.com/v2/r02/___https://grassrootsoccer.org/mental-health/___.YzJlOnVuaXNhbW9iaWxlOmM6b2ZmaWNlMzY1X2VtYWlsc19hdHRhY2htZW50OjQxNDNhNzEzNDZjMmZlMmViZjQ0ODBjNDgzOWE0MDc4Ojc6MTg0ZDpkNTI3YzljOGU0MjJlYmMyMTI5NWRiYWExNWFmZWEzOWVmOGYwYTI1ZGNmZmQxYzg1NWE3MmQ4MDYyYzAzOWFhOnA6VDpO)**)**

Through MindSKILLZ, a sport-based mental health program**,** Grassroot Soccer observed that the young people they support face unique challenges in accessing mental health support, largely due to linguistic and cultural barriers. Young people often face barriers which leave them feeling unwelcome in the mental health space. Such barriers include having their lived experience of chronic stress being dismissed with statements like “You’re too young to be stressed” from others in their communities and healthcare facilities.

Young people have developed their own coded language to discuss their challenges, avoiding conventional terminology that might create barriers to help-seeking. Rather than saying *"I'm depressed,*" *they might say* "*I'm going through the most*" or "*It's been rough*." This linguistic adaptation, while creative, can lead to prolonged periods without seeking help from a mental health professional for proper diagnosis or treatment.

Trained, near-peer mentor 'coaches' are a big part of Grassroot Soccer programmes, and they create young-people-friendly environments where young people can openly discuss their experiences without fear of stigma. Coaches are then able to identify and refer young people with acute mental health needs and social needs to appropriate mental health or child safeguarding professionals for further assessment and support.

Grassroot Soccer’s approach includes:

- Using accessible, age-appropriate language e.g. differences between stress and toxic stress.
- Supporting young people to build upon their existing skills, assets and strengths.
- Building support networks through peer engagement.
- Incorporating play to create fun, engaging learning environments and enhance social-emotional learning.
- Using near peer mentors from the same communities, facilitating personal testimony and shared experiences and language.
- Meaningfully engaging the young people at every point in the program life cycle – from design to implementation to evaluation.

The organization emphasizes the importance of early intervention and promoting wellbeing through simple age-appropriate language and creating safe spaces to encourage young people participation, while avoiding clinical labels that might deter young people participation. Grassroot Soccer’s approach demonstrates how mental health information and support can be effectively delivered through culturally sensitive, young-people-oriented programming that can bridge the gap between clinical services and young people's lived experiences.

1. **Real World Example 2: The work of Mind Matters in South Africa (**[**https://www.mindmattersnpo.org/**](https://protect.checkpoint.com/v2/r02/___https://www.mindmattersnpo.org/___.YzJlOnVuaXNhbW9iaWxlOmM6b2ZmaWNlMzY1X2VtYWlsc19hdHRhY2htZW50OjQxNDNhNzEzNDZjMmZlMmViZjQ0ODBjNDgzOWE0MDc4Ojc6ZDQ4Mjo1ZDA3OTkwMzAxYWJhMzY4N2ZjNmFmZjIxMTNjOTY2YTdlMmFlMmZmYzhmN2M0OWZmMzIwZDgyNjY5NDAxZmI0OnA6VDpO)**)**

In Mind Matters experience, young people in South Africa experience various barriers to accessing mental health support. These include individual, social, relationship as well as structural and systemic factors that can cause significant barriers to accessing mental health support. At an individual level, factors such as lack of knowledge about mental health challenges and how to access support as well as lack of trust and confidence in mental health professionals and effectiveness of interventions can create hesitation in accessing care. Young people can also be hindered by social and relationship factors where they perceive help-seeking as a sign of weakness, fear the negative public attitudes, and find it challenging talking to strangers (that being mental health professionals) about personal problems. Young people can also appear to prefer talking to people who are more familiar to them, which can further make “formalized” support services inaccessible. Moreover, structural and systemic factors such as high cost of care and remote location of mental health services, can further interfere with other activities, probably causing a significant barrier to help seeking.

Mind Matters is a non-profit organisation that strives to bridge this gap by providing support to individuals in taking brave steps towards improving their mental health and wellbeing, while inspiring their communities to do the same. We do this by implementing:

- Mental health literacy programmes.
- Impactful campaigns
- Capacity building in mental health support through lay counselling
- Mental health first aid training within schools.
- Task-shifting models (i.e. moving delivery of interventions from highly specialised mental health professions to support provided by trained non-mental health specialist members of society) to delivering mental health support.

Our approaches illustrate how young people can receive support within systems they are already embedded in within their communities, thereby helping in bridging the proximity and cost barrier to mental health support. Clients are assessed, supported, and referred forward for professional help based on the severity of their challenges and the resulting impact on social, occupational and other significant areas of functioning. We have observed that clients often respond positively to the focus on individual experiences and related challenges rather than attaching diagnostic labels that don’t give insight into the presenting problem and how it is linked to their current experiences. In our experience, the clients feel an increased sense of agency as they also gain insight into their individual experiences and some of the maintaining factors to these challenges. The focus of the lay counselling is capacity building for better management of these current and similar future challenges.

1. **The Radical Reconceptualization through the Ubuntu Lens**

Ubuntu philosophy (“I am because we are”) constructs personhood as fundamentally relational rather than autonomous [6, 8]. This presents an ontological challenge to diagnostic frameworks that locate distress within individuals. Where biomedical diagnostics ask, 'What disorder does this individual have?', Ubuntu frameworks ask, 'What relational disruption is this person expressing?' These are not equivalent questions and cannot be resolved through cultural sensitivity alone; they reflect different ontologies of personhood [6, 8]. This ontological distinction maps onto observable patterns in our contexts, where individual diagnostic labels operate on a category of selfhood that does not seem to reflect local experience. Traditional healers reframing psychotic symptoms as ancestral calling (ukuthwasa) are not rejecting diagnosis but correctly locating distress within relational-spiritual systems [8]. In these contexts, distress may be understood as an eco-relational signal, a disruption in the cohesiveness between the individual, their environment, and their broader community, rather than a psychological or biological disruption within a singular individual. Urban young people using “depression” colloquially to signal peer solidarity are navigating between individualized clinical language and relational experience. Without a biomedical conceptualisation of symptoms, people are less likely to seek care from formal health facilities [10]. This highlights that the individualised perspective on human challenges cannot be interpreted as neutral or as a universal objective truth. As Kpanake L [6] cautions, imposing individualized diagnostic categories “risks depriving some people of their social identity and amounts to possible erasure” p.6 [6]. Where Ubuntu personhood and individual identity are intertwined, the question must be asked: can an individual young person truly be 'disordered' in isolation?
